# Supplementary material for: Prognostic Value of the Immunohistochemical Detection of Cellular Components of the Tumor Microenvironment in Oral Squamous Cell Carcinoma: A Systematic Review
Source: Curr Issues Mol Biol. 2025 Jul 12;47(7):544. doi: 10.3390/cimb47070544 (PMC12293956; doi:10.3390/cimb47070544)
Supplement: Supplementary file 1 [file cimb-47-00544-s001.zip › Supplementary material S2.pdf]

Supplementary material S2. Search strategies used in this systematic review.

PubMed/Medline, Scopus, Embase, Cochrane, and Google Scholar

Search	Strategy
1#	(((“Oral cavity cancer” OR “oral cancer” OR Oral OR Carcinoma OR Carcinomas OR “Squamous Cell Carcinomas” OR “Squamous Cell Carcinoma” OR Epidermoid OR “Epidermoid Carcinoma” OR “Epidermoid Carcinomas”) AND (“Tumour microenvironment” OR Immune microenvironment OR T-cells OR B-cells OR CD4 OR CD8 OR dendritic cells OR endothelial OR Neutrophils OR Macrophage OR tumor-associated macrophage OR Cancer-associated fibroblasts OR CAF OR CAFS OR Regulatory T cells OR Tregs OR Natural-killer cells OR NK cells OR adipocytes OR adipocyte OR Macrophages OR dendritic OR endothelials OR Natural-killer) AND (Survival OR Mortality OR Prognosis OR Survival analysis OR Outcome OR Risk OR Risk ratio OR Relative risk OR Odds ratio OR Biomarker)))
2#	(((“Oral cavity cancer” OR tongue OR “oral cancer” OR Oral OR Carcinoma OR “Squamous Cell Carcinoma” OR Epidermoid OR “Epidermoid Carcinoma”) AND (T-cells OR B-cells OR CD4 OR CD8 OR Natural-killer cells OR NK cells OR Lymphocytes OR Regulatory T cells OR Tregs OR Th1 OR Th2) AND (Survival OR Mortality OR Prognosis OR Survival analysis OR Outcome OR Risk OR Risk ratio OR Relative risk OR Odds ratio OR Biomarker)))
3#	(((“Oral cavity cancer” OR tongue OR “oral cancer” OR Oral OR Carcinoma OR “Squamous Cell Carcinoma” OR Epidermoid OR “Epidermoid Carcinoma”) AND (Macrophage OR Macrophages OR M1 OR M2 OR CD68 OR CD163) AND (Survival OR Mortality OR Prognosis OR Survival analysis OR Outcome OR Risk OR Risk ratio OR Relative risk OR Odds ratio OR Biomarker)))
4#	(((“Oral cavity cancer” OR tongue OR “oral cancer” OR Oral OR Carcinoma OR “Squamous Cell Carcinoma” OR Epidermoid OR “Epidermoid Carcinoma”) AND (Neutrophils OR Neutrophil) AND (Survival OR Mortality OR Prognosis OR Survival analysis OR Outcome OR Risk OR Risk ratio OR Relative risk OR Odds ratio OR Biomarker)))
5#	(((“Oral cavity cancer” OR tongue OR “oral cancer” OR Oral OR Carcinoma OR “Squamous Cell Carcinoma” OR Epidermoid OR “Epidermoid Carcinoma”) AND (dendritic cells OR dendritic) AND

	(Survival OR Mortality OR Prognosis OR Survival analysis OR Outcome OR Risk OR Risk ratio OR Relative risk OR Odds ratio OR Biomarker)))
6#	(((“Oral cavity cancer” OR tongue OR “oral cancer” OR Oral OR Carcinoma OR “Squamous Cell Carcinoma” OR Epidermoid OR “Epidermoid Carcinoma”) AND (endothelial OR endothelial cell) AND (Survival OR Mortality OR Prognosis OR Survival analysis OR Outcome OR Risk OR Risk ratio OR Relative risk OR Odds ratio OR Biomarker)))
7#	(((“Oral cavity cancer” OR tongue OR “oral cancer” OR Oral OR Carcinoma OR “Squamous Cell Carcinoma” OR Epidermoid OR “Epidermoid Carcinoma”) AND (Cancer-associated fibroblasts OR CAF OR CAFS) AND (Survival OR Mortality OR Prognosis OR Survival analysis OR Outcome OR Risk OR Risk ratio OR Relative risk OR Odds ratio OR Biomarker)))
8#	(((“Oral cavity cancer” OR tongue OR “oral cancer” OR Oral OR Carcinoma OR “Squamous Cell Carcinoma” OR Epidermoid OR “Epidermoid Carcinoma”) AND (adipocytes AND adipocyte) AND (Survival OR Mortality OR Prognosis OR Survival analysis OR Outcome OR Risk OR Risk ratio OR Relative risk OR Odds ratio OR Biomarker)))

- Web of Science

Search	Strategy
1#	(((“Oral cavity cancer” OR tongue OR “oral cancer” OR Oral OR Carcinoma OR “Squamous Cell Carcinoma” OR Epidermoid OR “Epidermoid Carcinoma”) AND (adipocytes AND adipocyte) AND (Survival OR Mortality OR Prognosis OR Survival analysis OR Outcome OR Risk OR Risk ratio OR Relative risk OR Odds ratio OR Biomarker)))

- Science Direct

Search	Strategy
1#	(((Oral OR Carcinoma OR Epidermoid) AND (“Tumour microenvironment” OR Immune microenvironment) AND (Survival OR Prognosis OR Outcome OR Risk)))

2#	(((Oral OR Carcinoma OR Epidermoid) AND (T-cells OR B-cells OR CD4 OR CD8 OR Natural-killer cells OR Regulatory T cells))
3#	(((Oral OR Carcinoma OR Epidermoid) AND (Macrophage OR M1 OR M2 OR CD68 OR CD163))
4#	(((Oral OR Carcinoma OR Epidermoid) AND (Neutrophils OR Neutrophil) AND (Survival OR Prognosis OR Outcome OR Risk)))
5#	(((Oral OR Carcinoma OR Epidermoid) AND (dendritic cells OR dendritic) AND (Survival OR Prognosis OR Outcome OR Risk)))
6#	(((Oral OR Carcinoma OR Epidermoid) AND (endothelial OR endothelial cell) AND (Survival OR Prognosis OR Outcome OR Risk)))
7#	(((Oral OR Carcinoma OR Epidermoid) AND (Cancer-associated fibroblasts OR CAF OR CAFS) AND (Survival OR Prognosis OR Outcome)))
8#	(((Oral OR Carcinoma OR Epidermoid) AND (adipocytes AND adipocyte) AND (Survival OR Prognosis OR Outcome OR Risk)))
