# Supplementary material for: Prognostic Value of the Immunohistochemical Detection of Cellular Components of the Tumor Microenvironment in Oral Squamous Cell Carcinoma: A Systematic Review
Source: Curr Issues Mol Biol. 2025 Jul 12;47(7):544. doi: 10.3390/cimb47070544 (PMC12293956; doi:10.3390/cimb47070544)
Supplement: Supplementary file 1 [file cimb-47-00544-s001.zip › Supplementary material S3.pdf]

Supplementary material S3. Deleted articles and reasons for exclusion.

No.	Author and publication year.	Article title	Reason for exclusion
1	Ni et al. (2015)	Microlocalization of CD68+ tumor-associated macrophages in tumor stroma correlated with poor clinical outcomes in oral squamous cell carcinoma patients	Peripheral blood samples
2	Hu et al. (2016)	Tumor- associated macrophages correlate with the clinicopathological features and poor outcomes via inducing epithelial to mesenchymal transition in oral squamous cell carcinoma	Lip and oropharynx
3	Zhao et al. (2020)	Diminished CD68+ Cancer-Associated Fibroblast Subset Induces Regulatory T-Cell (Treg) Infiltration and Predicts Poor Prognosis of Oral Squamous Cell Carcinoma Patients	Does not correlate immunohistochemical markers of the tumor microenvironment with survival analysis
4	He et al. (2014)	CD163+Tumor-Associated Macrophages Correlated with Poor Prognosis and Cancer Stem Cells in Oral Squamous Cell Carcinoma	Lip and oropharynx
5	Wang et al. (2019)	Heat shock factor 1 in cancer-associated fibroblasts is a potential prognostic factor and drives progression of oral squamous cell carcinoma	Lip and oropharynx
6	Zhang et al. (2021)	Cancer-associated fibroblasts promote oral squamous cell carcinoma progression through LOX-mediated matrix stiffness	Multiplex immunohistochemistry or double staining
7	Guo et al. (2020)	Upregulation of CSF-1 is correlated with elevated TAM infiltration and poor prognosis in oral squamous cell carcinoma	Lip and oropharynx
8	Zhou et al. (2016)	CD19+IL-10+ regulatory B cells affect survival of tongue squamous cell carcinoma patients and induce resting CD4+ T cells to CD4+Foxp3+ regulatory T cells	Multiplex immunohistochemistry or double staining
9	Hanakawa et al. (2014)	Regulatory T-cell infiltration in tongue squamous cell carcinoma	Multiplex immunohistochemistry or double staining
10	Hayashi et al. (2022)	Tumor-infiltrating FoxP3+ T cells are associated with poor prognosis in oral squamous cell carcinoma	Lip and oropharynx
11	Wu et al. (2017)	Tumor-Infiltrating CD4+ Central Memory T Cells Correlated with Favorable Prognosis in Oral Squamous Cell Carcinoma	Multiplex immunohistochemistry or double staining
12	Wu et al. (2017)	Expression of VISTA correlated with immunosuppression and synergized with CD8 to predict survival in human oral squamous cell carcinoma	Does not correlate immunohistochemical markers of the tumor microenvironment with survival analysis

13	Moreira et al. (2010)	T regulatory cell markers in oral squamous cell carcinoma: Relationship with survival and tumor aggressiveness	Lip and oropharynx
14	Song et al. (2016)	Foxp3 overexpression in tumor cells predicts poor survival in oral squamous cell carcinoma	Lip and oropharynx
15	Zhou et al. (2018)	Density and location of CD3+ and CD8+ tumor-infiltrating lymphocytes correlate with prognosis of oral squamous cell carcinoma	Lip and oropharynx
16	Sun et al. (2018)	TGF-β1/TβRII/Smad3 signaling pathway promotes VEGF expression in oral squamous cell carcinoma tumor-associated macrophages	Lip and oropharynx
17	Kimura et al. (2019)	Macrophage CCL22 expression in the tumor microenvironment and implications for survival in patients with squamous cell carcinoma of the tongue	Does not correlate immunohistochemical markers of the tumor microenvironment with survival analysis
18	Ai et al. (2021)	CD68+ Macrophage Infiltration Associates With Poor Outcome of HPV Negative Oral Squamous Carcinoma Patients Receiving Radiation: Poly(I:C) Enhances Radiosensitivity of CAL-27 Cells but Promotes Macrophage Recruitment Through HMGB1	Does not correlate immunohistochemical markers of the tumor microenvironment with survival analysis
19	Marcus et al. (2004)	Prognostic factors in oral cavity and oropharyngeal squamous cell carcinoma - The impact of tumor-associated macrophages	Lip and oropharynx
20	Kang et al. (2018)	Macrophage migration inhibitory factor is a novel prognostic marker for human oral squamous cell carcinoma	Does not correlate immunohistochemical markers of the tumor microenvironment with survival analysis
21	Kimura et al. (2021)	Macrophage CCL22 expression promotes lymphangiogenesis in patients with tongue squamous cell carcinoma via IL-4/STAT6 in the tumor microenvironment	Does not correlate immunohistochemical markers of the tumor microenvironment with survival analysis
22	Supanimitjaroenporn et al. (2022)	Prognostic value of pretreatment lymphocyte-to-monocyte ratio in patients with advanced oral cavity cancer	Peripheral blood samples
23	Lu et al. (2017)	Melatonin represses oral squamous cell carcinoma metastasis by inhibiting tumor-associated neutrophils	Peripheral blood samples
24	Silva et al. (2018)	Immunohistochemical analysis of neutrophils, interleukin-17, matrix metalloproteinase-9, and neoformed vessels in oral squamous cell carcinoma	Does not correlate immunohistochemical markers of the tumor microenvironment with survival analysis
25	Gao et al. (2023)	Neutrophils regulate tumor angiogenesis in oral squamous cell carcinoma and the role of Chemerin	Peripheral blood samples
26	Hu et al. (2022)	Neutrophils Promote Tumor Progression in Oral Squamous Cell Carcinoma by Regulating EMT and JAK2/STAT3 Signaling Through Chemerin	Does not correlate immunohistochemical markers of the tumor microenvironment with survival analysis

27	Shinriki et al. (2014)	Stromal expression of neutrophil gelatinase-associated lipocalin correlates with poor differentiation and adverse prognosis in oral squamous cell carcinoma	Does not correlate immunohistochemical markers of the tumor microenvironment with survival analysis
28	Han et al. (2021)	Increased tumor-infiltrating plasmacytoid dendritic cells promote cancer cell proliferation and invasion via TNF-α/NF-κB/CXCR-4 pathway in oral squamous cell carcinoma	Does not correlate immunohistochemical markers of the tumor microenvironment with survival analysis
29	Xiao et al. (2019)	CD103+ T and Dendritic Cells Indicate a Favorable Prognosis in Oral Cancer	Multiplex immunohistochemistry or double staining
30	Liu et al. (2008)	Clinicopathologic significance of tumor cell-lined vessel and microenvironment in oral squamous cell carcinoma	Lip and oropharynx
31	Wen et al. (2023)	m6A modification-mediated BATF2 suppresses metastasis and angiogenesis of tongue squamous cell carcinoma through inhibiting VEGFA	Does not correlate immunohistochemical markers of the tumor microenvironment with survival analysis
32	Forootan et al. (1999)	Neoangiogenesis and squamous cell carcinoma of the tongue	Full text not available
33	Miyahara et al. (2007)	Tumor lymphangiogenesis correlates with lymph node metastasis and clinicopathologic parameters in oral squamous cell carcinoma	Does not correlate immunohistochemical markers of the tumor microenvironment with survival analysis
34	Haga et al. (2021)	Crosstalk between oral squamous cell carcinoma cells and cancer-associated fibroblasts via the TGF-β/SOX9 axis in cancer progression	Does not correlate immunohistochemical markers of the tumor microenvironment with survival analysis
35	Wang et al. (2023)	Cancer-associated fibroblasts in the invasive tumour front promote the metastasis of oral squamous cell carcinoma through MFAP5 upregulation	Does not correlate immunohistochemical markers of the tumor microenvironment with survival analysis
36	Saha et al. (2022)	Orchestrated expression of vasculogenic mimicry and laminin-5γ2 is an independent prognostic marker in oral squamous cell carcinoma	Lip and oropharynx
37	Kayamori et al. (2016)	NOTCH3 Is Induced in Cancer-Associated Fibroblasts and Promotes Angiogenesis in Oral Squamous Cell Carcinoma	Does not correlate immunohistochemical markers of the tumor microenvironment with survival analysis
38	Kyzas et al. (2005)	Evidence of lymphangiogenesis and its prognostic implications in head and neck squamous cell carcinoma	Lip and oropharynx
39	Marsh et al. (2011)	Stromal features are predictive of disease mortality in oral cancer patients	Lip and oropharynx
40	Nordfors et al. (2013)	CD8+ and CD4+ tumour infiltrating lymphocytes in relation to human papillomavirus status and clinical outcome in tonsillar and base of tongue squamous cell carcinoma	Lip and oropharynx

41	Wolf et al. (1986)	Lymphocyte subpopulations infiltrating squamous carcinomas of the head and neck: correlations with extent of tumor and prognosis.	Lip and oropharynx
42	Liu et al. (2022)	Tumor-Associated Macrophages Promote Metastasis of Oral Squamous Cell Carcinoma via CCL13 Regulated by Stress Granule	Does not correlate immunohistochemical markers of the tumor microenvironment with survival analysis
43	Haque et al. (2019)	CD206+ tumor-associated macrophages promote proliferation and invasion in oral squamous cell carcinoma via EGF production	Lymph nodes
44	Kai et al. (2021)	Oral Squamous Cell Carcinoma Contributes to Differentiation of Monocyte-Derived Tumor-Associated Macrophages via PAI-1 and IL-8 Production	Does not correlate immunohistochemical markers of the tumor microenvironment with survival analysis
45	Hu et al. (2016)	Tumor-associated macrophages correlate with the clinicopathological features and poor outcomes via inducing epithelial to mesenchymal transition in oral squamous cell carcinoma	Lip and oropharynx
46	Rani et al. (2020)	Clinicopathological correlation of tumor-stroma ratio and inflammatory cell infiltrate with tumor grade and lymph node metastasis in squamous cell carcinoma of buccal mucosa and tongue in 41 cases with review of literature	Does not correlate immunohistochemical markers of the tumor microenvironment with survival analysis
47	Stasikowska-Kanicka et al. (2018)	Immunohistochemical Analysis of Foxp3+, CD4+, CD8+ Cell Infiltrates and PD-L1 in Oral Squamous Cell Carcinoma	Does not correlate immunohistochemical markers of the tumor microenvironment with survival analysis
48	Ranieri et al. (2002)	Microvessel density, mast cell density and thymidine phosphorylase expression in oral squamous carcinoma	Does not correlate immunohistochemical markers of the tumor microenvironment with survival analysis
49	Santos et al. (2019)	Evaluation of Cd8+ and natural killer cells defense in oral and oropharyngeal squamous cell carcinoma	Lip and oropharynx
50	Kikuchi et al. (2006)	Vascular endothelial growth factor and dendritic cells in human squamous cell carcinoma of the oral cavity	Does not correlate immunohistochemical markers of the tumor microenvironment with survival analysis
51	Lucio et al. (2016)	Tumor-associated macrophages (TAMs): clinical-pathological parameters in squamous cell carcinomas of the lower lip	Lip and oropharynx
52	Caldeira et al. (2017)	Immunophenotype of neutrophils in oral squamous cell carcinoma patients	Does not correlate immunohistochemical markers of the tumor microenvironment with survival analysis
53	Kouketsu et al. (2023)	Myeloid-derived suppressor cells and plasmacytoid dendritic cells are associated with oncogenesis of oral squamous cell carcinoma	Lip and oropharynx
54	Patil et al. (2018)	Comparison of immunohistochemical expression of vascular endothelial growth factor and CD105 in oral squamous cell carcinoma: Its correlation with prognosis	Lip and oropharynx

55	Silveira et al. (2010)	Analysis of local immunity in squamous cell carcinoma of the tongue and lower lip	Lip and oropharynx
56	Chang et al. (2011)	Overexpression of macrophage inflammatory protein-3α in oral cavity squamous cell carcinoma is associated with nodal metastasis	Lip and oropharynx
57	Weber et al. (2015)	Macrophage polarisation changes within the time between diagnostic biopsy and tumour resection in oral squamous cell carcinomas—an immunohistochemical study	Does not correlate immunohistochemical markers of the tumor microenvironment with survival analysis
58	Fang et al. (2015)	Elevated S100A9 expression in tumor stroma functions as an early recurrence marker for early-stage oral cancer patients through increased tumor cell invasion, angiogenesis, macrophage recruitment and interleukin-6 production	Does not correlate immunohistochemical markers of the tumor microenvironment with survival analysis
59	Arora et al. (2005)	Stromelysin 3, Ets-1, and Vascular Endothelial Growth Factor Expression in Oral Precancerous and Cancerous Lesions: Correlation with Microvessel Density, Progression, and Prognosis	Lip and oropharynx
60	Li et al. (2013)	Expression of Angiopoietin-2 and Vascular Endothelial Growth Factor Receptor-3 Correlates with Lymphangiogenesis and Angiogenesis and Affects Survival of Oral Squamous Cell Carcinoma	Multiplex immunohistochemistry or double staining
