# Supplementary material for: Prognostic Value of the Immunohistochemical Detection of Cellular Components of the Tumor Microenvironment in Oral Squamous Cell Carcinoma: A Systematic Review
Source: Curr Issues Mol Biol. 2025 Jul 12;47(7):544. doi: 10.3390/cimb47070544 (PMC12293956; doi:10.3390/cimb47070544)
Supplement: Supplementary file 1 [file cimb-47-00544-s001.zip › Supplementary material S4.pdf]

Supplementary material S4. Summary of descriptive characteristics, analysis methods and results of included studies that evaluated Cancer-Associated Fibroblasts.

Author, publication year and country	Sample size	Sublocation of oral cavity tumor	Sex	Age	TNM staging	Local recurrence	Death	Histological grading (WHO)	Follow-up	Biomarker used	IMH analysis method	Conclusions/Main results	Compliance to REMARK guidelines	Risk of Bias (MAStARI)
Kellermann et al.[57] Brazil	38	14 (tongue); 7 (floor of mouth); 7 (palate); 5 (buccal mucosa); 5 (gingiva)	34 (M); 4 (F)	61.1 years (mean)	4 (I); 9 (II); 11 (III); 14 (IV)	11	28	14 (well differentiated); 18 (moderately differentiated); 6 (poorly differentiated)	NI	α-SMA	The presence of a-SMApositive cells was classified as negative (0), scanty (1) or abundant (2) in a blinded analysis.	The results of the study suggest that during tumor invasion OSCC-derived TGFb1 promote fibroblast–myofibroblast transdifferentiation, and that tumor cellular proliferation can be induced by factors released from myofibroblasts, which may favor tumor growth.	Checklists no. 4, 5 and 6 were not fulfilled	High
Vered et al.[53] Israel	50	Tongue	26 (M); 24 (F)	57.9 years (mean)	33 (I/II); 17 (III/IV)	17	16	NR	46.7 months (mean)	α-SMA	Quantitatively, the frequency of Stromal myofibroblasts was assessed on a 5-scale scoring system: 0, devoid of Stromal myofibroblasts; 0.5, a few Stromal myofibroblasts with a spindle-shaped morphology tightly adhering to the periphery of the SCC islands/nests; 1, Stromal myofibroblasts surround the tumor in a few concentric layers in several foci; 2, Stromal myofibroblasts with both spindle-shaped and plump morphology in many areas of the tumor; and 3, similar to the former score but Stromal myofibroblasts were exceptionally abundant throughout the section, occasionally exceeding the carcinomatous component.	It seems that abundant stromal myofibroblasts (camouflaging some malignant cells) and high-risk scores have an unfavorable impact on the risk of recurrence in particular in ‘young’ patients.	All checklists were completed	Low
Bello et al.[43] Finland	128	Tongue	60 (M); 68 (F)	65 years	70 (I/II); 55 (III/IV); 3 (NI)	47	61	NR	54 months (mean)	α-SMA	The density of CAFs (alfa-SMA +) in the TME (score poor/medium/rich).	CAF-rich TME being associated with increased mortality from the disease itself.	Checklist no. 5 was not fulfilled	High

Dayan et al.[50] Israel	64	Tongue	33 (M); 31 (F)	57.4 years (M); 65 years (F)	15 (I/II); 49 (III/IV)	NI	NI	NR	63 years (mean)	TGF-b, α-SMA	Assessment of the immunostains was performed semiquantitatively on a scale of 0 to 5, where 0 = no staining, 1 = staining of weak intensity in <50% cells, 2 = weak but extensive (>50% cells) staining, 3 = strong staining in <50% cells, and 4 = strong staining in >50% cells.	The overall density of the inflammatory infiltrate was inversely correlated to the density of CAFs (P = 0.01), but that the cumulative density of the protumorigenic/anti-inflammatory phenotypes was directly correlated with the density of CAFs (P = 0.01).	Checklist no. 2 was not fulfilled	Moderate
Fujii et al.[13] Japan	108	41 (tongue); 33 (lower gingiva); 15 (upper gingiva); 10 (floor of the mouth); 6 (buccal mucosa); 3 (palate)	67 (M); 41 (F)	66.4 years (mean)	23 (I); 23 (II); 11 (III); 51 (IV)	NI	NI	82 (well/ moderate differentiated; 26 (poor differentiated)	120 months	α-SMA	4 grades on the basis of staining intensity: negative (0), scanty (1), focal (2), and abundant (3)	CAFs are possible prognostic factors of OSCC. We confirmed the presence of CAFs in early to advanced stage OSCC and consider that CAFs may be associated with cancer invasion.	Checklist no. 4 was not fulfilled	Moderate
Ding et al.[40] China	50	Tongue	25 (M); 25 (F)	53.5 years (mean)	25 (I/II); 25 (III/IV)	21	19	28 (I) 20 (II) 2 (III)	60.34 months (mean)	α-SMA	Four grades: 0 - 3. The immunoreactivity was divided into three groups: grades 0 and 1 were grouped as negative immunoreactivity, grade 2 as low, grade 3 as high. The score from 0 to 1 means a-SMA-poor; the score 2, medium, and the score 3, rich.	a-SMA-positive myofibroblasts have important impacts on cancer progression, metastasis, and survival prognosis of patients with OTSCC.	All checklists were completed	Low
Li et al.[42] China	178	Tongue	106 (M); 72 (F)	88 (< 60 years); 90 (≥ 60 years)	82 (I); 64 (II); 17 (III); 15 (IV)	60	57	NR	78.3 months (mean)	α-SMA	Quantitatively, the number of CAFs was assessed on a 5-scale scoring system: 0, devoid of CAFs; 0.5, a few CAFs with a spindle-shaped morphology that adhered closely to the periphery of the SCC islands/nests; 1, CAFs surrounded the tumor in a few concentric layers in several foci; 2, CAFs with both a spindle-shaped and a plump morphology were observed in many areas of the tumor; 3, similar to a score of “2”, but CAFs were	CAFs were an independent prognostic factor for patients with OTSCC.	Checklists no. 2 and 5 were not fulfilled	High

											exceptionally abundant throughout the section and occasionally exceeded the carcinomatous component.			
Luksic et al.[71] Croatia	152	72 (floor of the mouth); 43 (tongue); 22 (retromolar space); 15 (lower gingiva)	124 (M); 28 (F)	59 years (mean)	NI	20	34	72 (well differentiated); 43 (moderately differentiated); 37 (poorly differentiated)	6 months (minimum)	α-SMA	The proliferation of myofibroblasts within the tumour stroma was assessed semiquantitatively with a 5-point scoring system: 0 = no myofibroblasts, 1 = present in up to 25% of the stroma, 2 = present in 26–50% of the stroma, 3 = present in 51–75% of the stroma, and 4 = present in more than 76% of the stroma. For the statistical analysis, cases with low scores (0 and 1) were combined and compared to cases with high scores (2, 3, and 4).	In conclusion, this study demonstrated that an abundant presence of myofibroblasts leads to a more aggressive phenotype of the OSCC, resulting in a significantly increased presence of occult metastases, regional recurrence, and distant metastases, as well as lower survival rates. The multivariate model derived from this study identified a high myofibroblast score as an independent predictor of occult neck disease, thus identifying those patients most likely to benefit from elective neck dissection. Therefore, myofibroblasts in oral cancer facilitate tumour growth and metastatic spread.	Checklists no. 2, 4 5 and 6 were not fulfilled	Moderate
Matsuoka et al.[45] Japan	60	19 (tongue); 10 (mandible); 12 (maxilla); 9 (oral floor); 10 (buccal mucosa).	36 (M); 24 (F)	68.9 years (mean)	6 (II); 21 (III); 33 (IV)	NI	NI	46 (well differentiated); 14 (moderate differentiated)	90 months (mean)	α-SMA	One score was assigned according to the percentage of positive cells: <10%: 1 point; 10–50%: 2 points; >51%: 3 points and another score was assigned according to the intensity of the staining, with negative to weak staining equaling 1 point, moderate staining equaling 2 points and strong staining equaling 3 points. If the expression score was ≥4, the tumour was thus considered to have a high expression.	A Kaplan–Meier analysis revealed that higher numbers of CAFs and TAMs were significantly correlated with a poor prognosis.	All checklists were completed	Low
Attramadal et al.[52] Norway	62	13 (gingiva); 17 (tongue); 25 (floor of mouth); 7 (other sites)	40 (M); 22 (F)	61 years (mean)	62 (I/II)	15	NI	NI	55 months (mean)	α-SMA	The CAFs were grouped into low, moderate and high expression.	Receiving postoperative radiotherapy when having a high CAF density at the invasive front was apparently associated with increased recurrence.	Checklists no. 2, 4, 5 and 6 were not fulfilled	High

Lao et al.[46] China	93	Tongue	56 (M); 37 (F)	52 years (mean)	63 (I/II); 13 (III/IV)	NI	25	47 (well differentiated); 40 (moderately differentiated); 6 (poorly differentiated)	41 months (median)	α-SMA	Immunostaining of CAFs was assessed by evaluation of the staining intensity and percentage of α-SMA. The percentage of immunopositive CAFs in non-inflammatory and non-endothelial stromal cells was recorded as follows: 0, no positive cells; 1, 1–33% positive cells; 2, 34–66% positive cells; and 3, 67–100% positive cells. Staining intensity was considered to be 0 when there was no staining, 1 where positivity was observed only at magnification, ×400, 2 in cases where staining was evident at ×200 but not at ×100 and 3 in fields where immunopositive cells were observed even at magnification, ×100. Multiplication of the percentage and intensity scores composed the staining index of each specimen. This index was classified as negative (score 0), low (score 1–2), moderate (score 3–4) or high (score 6–9).	The degree of CAFs/interstitial collagen fibers correlates with the T stage of TSCC. Distribution patterns of interstitial fibrosis are associated with overall survival of TSCC patients. A pattern of high interstitial fibrosis correlated with the lowest overall survival rate. The results of the present study may assist with additional investigation of inflammation and interstitial fibrosis in the TSCC microenvironment.	Checklists no. 4, 5 and 6 were not fulfilled	High
Dourado et al.[47] Brazil	93	45 (tongue); 48 (others)	70 (M); 23 (F)	60 years (mean)	70 (I/II); 55 (III/IV); 3 (NI)	34	43	65 (well differentiated); 18 (moderately differentiated); 10 (poorly differentiated)	123 months	α-SMA	Tumors were classified as negative if 0% of the fibroblasts were α -SMA positive, scanty if 1% to 50% were α -SMA positive, and abundant if >50% of the stromal fibroblasts were α -SMA positive.	Associations of clinical stage with DSS and CAF density with DFS were found.	All checklists were completed	Low
Liang et al.[44] China	26	NI	21 (M); 5 (F)	12 (<50 years); 14 (≥50 years)	NI	5	NI	NI	16.19 months (mean)	α-SMA	Immunostaining of CAFs was investigated by evaluating the staining intensity (0, negative; 1, weak; 2, moderate; and 3, strong) and percentage (0%–100%) of α-SMA in non-inflammatory and non-endothelial stromal cells using a similar	This study showed that high p62 expression of OSCC cells statistically correlated with stromal α-SMA expression after chemotherapy. CAFs increased after chemotherapy. Further studies are needed to verify the relation	Checklists no. 1, 2 and 5 were not fulfilled	High

											evaluation system. The final expression score for CAFs was calculated by multiplying the percentage by the intensity score.	between CAFs and cancer cell autophagy.		
Vered et al.[41] Israel	54	Tongue	NI	NI	NI	NI	NI	NI	NI	α-SMA	Was assessed in 5 randomly selected high power fields within the tumor area and scored as 1 – one-to-five stained cells in each field, 2 – more than 5 stained cells in each field; any finding less than score 1, was allocated a score of 0. Results are presented as poor, intermediate and rich.	CAFs are negative prognosticators in tongue cancer.	Checklists no. 2 and 5 were not fulfilled	High
Qiu et al.[18] China	100	28 (buccal); 58 (tongue); 14 (gingival)	63 (M); 37 (F)	57.08 years (mean)	32 (I); 26 (II); 18 (III); 24 (IV)	NI	NI	35 (I) 48 (II) 17 (III)	30.33 months (mean)	FAP, α-SMA	Patients were categorized into three groups according to CAFs–stroma ratio: low CAFs group (CSR <33%), medium CAFs group, and high CAFs group (CSR >66%).	The close relation was revealed between CSR and TSR in the prognosis based on the evaluation of the immunohistochemical results and indicating the critical role of CAFs in the tumor development.	All checklists were completed	Low

Legends: UK: United Kingdom; USA: United States of America; M: male; F: female; NI: not informed; NR: not realized; RT: radiotherapy.
