# Supplementary material for: Prognostic Value of the Immunohistochemical Detection of Cellular Components of the Tumor Microenvironment in Oral Squamous Cell Carcinoma: A Systematic Review
Source: Curr Issues Mol Biol. 2025 Jul 12;47(7):544. doi: 10.3390/cimb47070544 (PMC12293956; doi:10.3390/cimb47070544)
Supplement: Supplementary file 1 [file cimb-47-00544-s001.zip › Supplementary material S5.pdf]

Supplementary material S5. Summary of descriptive characteristics, analysis methods and results of included studies that evaluated immune microenvironment.

Author, publication year and country	Sample size	Sublocation of oral cavity tumor	Sex	Age	TNM staging	Local recurrence	Death	Histological grading (WHO)	Follow-up	Biomarker used	IMH analysis method	Conclusions/Main results	Compliance to REMARK guidelines	Risk of Bias (MAStARI)
Goldman et al.[38] USA	43	Tongue	23 (M); 20 (F)	61 years (mean)	NI	NI	NI	NR	5 years (minimum)	DC: CD1a e S100	Counts were obtained in 5 high-power fields (×400 magnification). Dendritic cells were counted in areas of greatest staining intensity.	The association between CD1a-positive peritumoral DCs and patient outcome suggests an important function for this cell population.	Checklists no. 2 and 5 were not fulfilled	Moderate
Reichert et al.[37]	132	56 (floor of the mouth); 15 (maxilla, including the palate); 7 (retromolar trigone); 24 (gingiva of the mandible); 5 (buccal mucosa); 25 (tongue)	101 (M); 31 (F)	58 years (median)	30 (I); 32 (II); 7 (III); 63 (IV)	NI	58	16 (I); 89 (II); 27 (III)	50 months (median)	DC: S100	To determine DC numbers, at least 10 high-power fields (HPF) were selected randomly for microscopic examination (at ×400 magnification) at the border of the tumor and within the peritumoral tissue. DCs were counted only when the cell nuclei could be identified; dendritic processes were not counted. The number of DCs positive for S-100 per 10 HPFs was determined for every specimen. The, specimens were divided into three groups distinguishable by low density of DC infiltrate (<10 cells per HPF), intermediate density of DC infiltrate (11–20 cells per HPF), and high density of DC infiltrate (>20 cells per HPF).	The number of DCs infiltrating the tumor is a highly significant prognostic parameter in patients with OSCC.	Checklists no. 4 and 5 were not fulfilled	Moderate
O'Donell et al.[34] USA	63	NI	46 (M); 17 (F)	35 (≤ 60 years); 28 (> 60 years)	NI	NI	22	NR	4.8 years (median)	DC immature: CD207, CD209/ DC plasmocitoid: CD123	Slides were scored blind to outcome. For each antibody, slides were scored semiquantitatively into four groups: 0, 1+, 2+, and 3+ based on approximate numbers of stained cells.	Although the majority of DC-SIGN+ immature dermal DCs were present outside the tumor margin, their presence intratumorally was significantly associated with decreased survival. The presence of CD123+ plasmacytoid dendritic cells (pDCs) was also	Checklists no. 2, 3, 4 and 5 were not fulfilled	High

												significantly associated with decreased survival.		
Zancope et al.[19] Brazil	40	41 (oral tongue); 33 (floor of the mouth); 26 (others)	64 (M); 36 (F)	58 (≤60 years); 42 (>60 years)	NI	NI	NI	38 (I/II); 62 (III/IV)	NI	T cells: CD8 / NK cells: CD57	The density (per mm2) and the percentage (in relation to the total of the inflammatory cells) of CD8 and NK cells in the stroma near the invasion front of oral SCC (peritumoral) were determined. In addition, the numbers of CD8 and NK cells within the cancer nests (intratumoral) or in the intraepithelial region were also evaluated. All counts were performed in 10 alternate microscopic high-power fields (×400).	The differential CD8 and NK cells infiltration in oral SCC might reflect a distinctive tumor microenvironment with a favorable local cytotoxic immune response against neoplastic cells.	Checklists no. 2 and 5 were not fulfilled	Moderate
Watanabe et al.[20] Japan	87	40 (gingiva); 37 (tongue); 10 (others)	52 (M) 35 (F)	65 years (mean)	17 (I); 38 (II); 10 (III); 22 (IV)	NI	NI	28 (well differentiate d); 45 (moderately differentiate d); 14 (poorly differentiate d)	NI	T cells: CD8, CD4, CD25, CD69 / Treg: Foxp3,	Photographs were obtained from 30 randomly selected areas within the tumor–host interface (30 areas were composed of 15 cancer nest areas and 15 cancer stroma areas) at a high-power magnification (×100 objective) in all 87 cases. Positive cells were counted manually using the enlarged photographs. the results were averaged.	Host immune responses in the stroma of OSCC affect the survival of the patients. The in situ balance between effector T cells and regulatory T cells is the most important factor predicting survival.	Checklist no. 5 was not fulfilled	High
Cho et al.[69] Korea	45	NI	32 (M); 13 (F)	20 (<59 years); 25 (⩾59 years)	25 (I/II); 20 (III/IV)	19	NI	33 (well differentiate d); 12 (moderately differentiate d)	125 months	T cells: CD8, CD4	The numbers of CD4+ and CD8+ lymphocytes within the tumor cell nests (intratumoral) and in the stroma near the invasive front of OSCC (peritumoral) were counted. The mean value of five fields was used in the statistical analysis. The area of one field was 0.066 mm2. The intensity of positive TAFs was graded as 0 (none), 1 (weak), 2 (moderate), and 3 (strong). The distribution was scored as 0, no stained cells; 1, 1– 50%; and 2, 51–100% stained cells. Each a case was considered positive when the sum of distribution	Peritumoral TILs were significantly associated with tumor size, lymph node metastasis and clinical stage. However, the densities of peritumoral TILs or their ratios were not correlated with patient survival.	Checklists no. 4, 5 and 6 were not fulfilled	Moderate

											and intensity scores (range, 0–5) was 3 or higher.			
Liang et al.[25] China	81	Tongue	45 (M); 36 (F)	27 (<50 years); 54 (⩾50 years)	39 (I/II); 42 (III/IV)	18	NI	36 (well differentiated); 27 (moderately differentiated); 18 (poorly differentiated)	NI	Treg: Foxp3	Staining of at least 25% of the TSCC cells was considered positive for Foxp3 expression.	Foxp3 expression in TSCC cells was an independent prognostic indicator for TSCC.	All checklists were completed	Moderate
Dayan et al.[50] Israel	64	Tongue	33 (M); 31 (F)	57.4 years (M); 65 years (F);	15 (I/II); 49 (III/IV)	NI	NI	NI	63 years (mean)	Machophages: CD80 / B cell: CD138 / Treg: Foxp3	Assessment of the immunostains was performed semiquantitatively on a scale of 0 to 5, where 0 = no staining, 1 = staining of weak intensity in <50% cells, 2 = weak but extensive (>50% cells) staining, 3 = strong staining in <50% cells, and 4 = strong staining in >50% cells.	Univariate analysis demonstrated that a high Foxp3 score had a negative impact on recurrence (P = 0.026), while the density of the inflammatory infiltrate as well as the other individual types of inflammatory cells had no impact on either recurrence or patient survival (P > 0.05).	Checklist no. 2 was not fulfilled	Moderate
Fujita et al.[70] Japan	50	17 (tongue); 7 (upper gingiva); 16 (lower gingiva); 6 (buccal mucosa); 3 (floor of the mouth); 1 (mandibular boné)	32 (M); 18 (F)	68,6 years (mean)	9 (I); 18 (II); 4 (III); 19 (IV)	NI	NI	40 (well differentiated); 8 (moderate differentiated); 2 (poor differentiated)	NI	Treg: Foxp3 /Macrophage: CD163	Quantitative studies of the immunohistochemically stained sections were performed by pathologists in a blind fashion by evaluating three randomly chosen fields in each sample. Individual cells were counted under microscopic fields. The cells stained with anti-IL-8 antibody were counted on tumor cells (IL-8(T)) and on stromal cells (IL-8(S)) in tumor tissues, and the cases in which over 5% of the cells were stained, were defined as positive expression (+). We counted the numbers of Foxp3- or CD163-stained immune cells that had infiltrated into the tumor (Foxp3(IT) or CD163(IT)) and those that had infiltrated the tumor invasive front (Foxp3(IF) or CD163(IF)) by using BIOREVO BZ-9000 (Keyence, Elmwood	The density of CD163-positive cells at the invasive front of the tumor, i.e., CD163(IF) was associated with a poor clinical outcome (p = 0.002, in DFS) in all patients. A multivariate analysis revealed that CD163(IF) status significantly affected the DFS of patients.	Checklist no. 5 was not fulfilled	Moderate

											Park, NJ), and each median value was chosen as the cut-off value.			
Ni et al.[35] China	79	NI	38 (M); 41 (F)	46 (<60 years); 33 (≥60 years)	45 (I/II); 34 (III/IV)	NI	19	32 (well differentiated); 47 (medium to poor differentiated)	NI	mature DC: CD208	The number of cells with an mDC morphology was counted. The number of CD208+ cells was calculated in 5 representative high-power fields (×400) in each tumor stroma (TS), the tumor nest (TN), and adjacent non-neoplastic tissues (NTs) sample.	The accumulation of CD208+ mDCs does not correlate with survival time in oral squamous cell carcinoma patients.	Checklists no. 4 and 5 were not fulfilled	Moderate
Sakakura et al.[49] Japan	74	Tongue	27 (M); 47 (F)	69 years (mena)	47 (I/II); 27 (III/IV)	27	22	66 (well-differentiated/Moderately differentiated); 8 (poorly differentiated)	133 months (maximum)	DC: CD1a / T cell: CD3 / NK cell: CD56	More than five areas of a representative field were counted at ×200 magnification for CD1a+ DC and CD56+ NK cells. The infiltration of CD3+ T cells in more than five ×400 high power fields (HPF) was graded as grade 1 (<10 positive cells/HPF), grade 2 (10–30/HPF), grade 3 (31–100/HPF) and grade 4 (>101/HPF), which was consistent with a previous study.	We also examined the relationship between overall/progression-free survival rates and the number (divided into higher/lower groups by median) of CD1a+ dendritic cells, CD3+ T cells or CD56+ NK cells,; however, no correlations were detected among these parameters.	Checklists no. 4, 5 and 6 were not fulfilled	Moderate
Hilly et al.[31] Israel	18	Tongue	11 (M); 7 (F)	64 years (mean)	NI	5	NI	18 (well or moderately differentiated)	61 months (mean)	Dendritic cells: S100, CD1a	Counts were performed in 10 high-power fields (HPFs)/case in tumoral tissue and in normal peritumoral tissue (Olympus BX50, UPlanFl 40×, ocular magnification ×10; area of microscopic field, 0.55 mm²).	Better disease-free survival was associated with low peritumoral S100- and CD1a- positive cell counts, and with low tumoral S100- and CD1a- positive cell counts.	Checklists no. 2 and 5 were not fulfilled	Moderate
Lao et al.[46] China	93	Tongue	56 (M); 37 (F)	52 years (mean)	63 (I/II); 13 (III/IV)	NI	25	47 (well-differentiated); 40 (moderately differentiated); 6 (poorly differentiated)	41 months (median)	B cells: CD19	The lesion cores were initially scored by assessing the proportion of each core that comprised epithelia and stroma at low magnification, and subsequently scoring the number of positively stained CD19+ B cells within the core area (by direct counting up to 20, or by estimation to the nearest 10 when >20) at magnification, ×200. Cores were scored as 0 (no cells present), 1 (1–5 cells), 2 (6–19 cells) or 3 (≥20 cells).	CD19+ B cells are associated with overall survival of TSCC patients. Low levels of CD19+ B cells correlated with the lowest overall survival rate.	Checklists no. 4, 5 and 6 were not fulfilled	High

Fang et al.[24] China	78	NI	57 (M); 21 (W)	60 years (mean)	36 (I/II); 42 (III/IV)	NI	NI	57 (well differentiated); 21 (moderate or poor differentiated)	48 months (median)	T cells: CD8, CD4; Macrophage: CD68; NK cells: CD57	The images were semi-automatically evaluated using the image analysis software COUNT (Biomas, Erlangen, Germany). For each section, 10 areas of a representative field of tumor were assessed using an ocular grid comprising a high-power field (HPF) area of 0.0314 mm2. Tumor areas were divided into three anatomic compartments (i.e. tumor epithelial, tumor stroma and advancing tumor margin). The total number of each type of immune cells in tumor stroma, excluding cells within tumour cell nests, was counted. The average number of 10 HPFs was calculated as the final density of each section (cells per hpf).	The univariate COX regression analyses showed that higher CD8 and CD57 expression ($p < 0.001$) were positively correlated with longer overall survival. Multivariate COX regression analysis showed that higher CD8 ($p = 0.03$) and CD57 ($p < 0.001$) expression could be independent prognostic indicators of better survival. None of CD4, T-bet or CD68 was associated with survival in ether univariate or multivariate analysis.	All checklists were completed	Moderate
Han et al.[33] China	60	26 (tongue); 5 (mouth floor); 12 (gingival); 13 (buccal mucosa); 4 (hard palate)	38 (M); 22 (F)	24 (<50 years); 36 (≥50 years)	NI	NI	NI	33 (well differentiated); 27 (moderate/ poorly differentiated)	34.5 months (mean)	Plasmacytoid dendritic cells (pDCs): CD123	pDC infiltrates were considered low if <10 pDCs per HPF (high-power field) were observed and high if >10 pDCs per HPF were seen.	The increased number of tumor-infiltrating pDCs correlates with an adverse outcome in primary OSCC patients.	Checklists no. 2 and 5 were not fulfilled	High
Jardim et al.[32] Brazil	53	37 (tongue); 16 (floor of the mouth)	40 (M); 13 (F)	56 years (mean)	7 (I); 7 (II); 23 (III); 16 (IV)	33	34	25 (well differentiated); 28 (moderately/ poorly differentiated)	NI	Dendritic cells: CD1a, CD83	The staining intensity was classified as strongly positive (red), positive (orange), weakly positive (yellow) or negative (blue). For counting, we considered valid only the stains with red (strongly positive) and orange (positive). We have selected 5 fields of 1 mm2 for both, intratumoral and peritumoral, and the results were expressed as the mean positive count staining of the 5 areas analyzed, that were translated into the density of cells per mm².	The depletion of peritumoral CD1a+ cells is an independent factor associated with overall survival and disease-free survival.	All checklists were completed	Low
Mafra et al.[56] Brazil	56	Tongue	39 (M); 17 (F)	62 years (mean)	20 (I/II); 36 (III/IV)	14	28	NR	NI	Mast cells: Triptase	Mast cells (tryptase-positive) were identified as cells exhibiting brown cytoplasmic staining, irrespective of the	There were no differences statistically significant with tumor size, local	Checklists no. 4, 5 and 6 were not fulfilled	Moderate

											intensity of staining. Five microscopic fields in the intra- and peritumoral areas containing the largest number of immunostained cells were photographed (scale = 100 μm) and mast cells were quantified in each area (scale = 50 μm). Mast cell density (MCD) was defined as the mean number of mast cells per intratumoral (I-MCD) or peritumoral (P-MCD) microscopic field.	recurrence, clinical outcome, histological risk score, lymphocytic infiltrate or perineural invasion ($p > 0.05$).		
Wirsing et al.[51] Norway	75	36 (tongue); 21 (floor of the mouth); 9 (alveolar ridge); 7 (buccal mucosa); 2 (others)	43 (M); 32 (F)	28 (0-59 years); 47 (≥ 60 years)	NI	NI	NI	28 (well differentiated); 42 (moderate differentiated);5 (poor differentiated)	60 months (maximum)	T cells: CD3, CD4, CD8 / B cells: CD20 /Macrophages: CD68; DC: LAMP/CD208	Two trained, independent observers who were blinded to the clinical outcome evaluated the immunohistochemical staining quantitatively and semiquantitatively.	In univariate analysis, high levels of CD20+ B cells and CD68+ macrophages, positive high-endothelial venule status, and low T and N stages predicted longer patient survival. However, they lost a significant association in the multivariate analysis.	All checklists were completed	Low
Boxberg et al.[55] Germany	66	NI	42 (M); 24 (F)	63 years (mean)	24 (I/II); 42 (III/IV)	NI	NI	7 (well-differentiated); 42 (moderately differentiated); 17 (poorly differentiated)	50 months (mean)	T cells: CD3+, CD8+, CD4+ / Treg: FOXP3+	The analysis of TIL subpopulations was performed in two ways: 1) Intraepithelial TILs: the tumor region of the respective cores showing the highest density of the particular TIL subpopulation was selected on low-power magnification (×4). Within this region, the amount of intraepithelial CD3+ TILs (CD3i), intraepithelial CD8+ TILs (CD8i), and intraepithelial FOXP3+ TILs (FOXP3i) was scored manually by counting the absolute number of TILs within TC clusters of 100 TCs using high-power magnification (×40; in analogy to Ref. 47). 2) Stromal TILs: In analogy to previous TIL-scoring approaches, density of	Patients with FOXP3 elevated carcinomas had a significantly more favorable prognosis.	Checklist no. 2 was not fulfilled	Low

											TILs was evaluated via determination of the percentage of the tumor stromal area occupied by the respective TIL subpopulations (CD3s; CD8s; FOXP3s). Corresponding tumor cores of each case and location were analyzed, and the average density across all cores of one specific region was calculated.			
Shimizu et al.[21] Japan	139	90 (tongue/floor of the mouth); 49 (others)	77 (M); 62 (F)	67 years (mean)	42 (I); 61 (II); 36 (III-IV)	NI	NI	52.5% (I); 43.9% (II); 4.6% (III)	5 years	T cells: CD8	The density of CD8+ T cells was assessed from 5 compartments: stroma in the center of the tumor; parenchyma in the center of the tumor; stroma at the invasive edge of the tumor; parenchyma at the invasive edge of the tumor; and tumor periphery. For the evaluation of tumor-infiltrating CD8+ T cell density in each compartment, at least three random fields were examined, and in cases of heterogeneity, the most representative count for the entire section was assigned. The average density of T cells for each compartment was used to stratify patients into high and low CD8+ T cell density groups.	Cox regression analysis revealed that high stromal CD8+ T-cell density at the tumor periphery and high parenchymal CD8+ T-cell density at the invading edge were independent prognostic makers (hazard ratio: 0.38 and 0.19, 95% confidence interval, 0.18-0.80 and 0.05-0.72, P = 0.01 and 0.01, respectively) for RFS and OS, respectively.	All checklists were completed	Low
Huang et al.[26] Taiwan	258	Tongue	222 (M); 36 (F)	52.7 years (mean)	24% (I); 24.8% (II); 16.7% (III); 34.5% (IV)	NI	NI	NR	4.5 years (mean)	T cells: CD3 and CD8	Images were captured in four different areas with the highest densities of CD3+ and CD8+ cells along the invasive margin under 200X magnification. The numbers of CD3 + and CD8 + TILs were identified using ImagePro software and then averaged for each slide.	Univariate analysis revealed that lower CD8 count was associated with shorter overall survival.	All checklists were completed	Low
Koike et al.[63] Japan	137	89 (tongue/floor of the mouth); 48 (other)	76 (M); 61(F)	63 (<68 years); 74 (≥ 68 years)	42 (I); 61 (II); 34 (III/IV)	16	NI	73 (I); 59 (II); 5 (III)	79 months (mean)	Treg: Foxp3	FoxP3+ T-cells and CTLA-4+ cells were evaluated using four different areas, including the parenchyma and stroma at the tumor center	Five-year overall survival, disease-specific survival, and recurrence-free survival were favorable in patients	All checklists were completed	Low

											(TCe), and the parenchyma and stroma at the invasive front (IF). First, FoxP3+ T-cells and CTLA-4+ cells were identified under 40× magnification; then, FoxP3+ T-cells and CTLA-4+ cells in the four regions of the tumor were counted visually. For counting, we chose the areas with the most intense FoxP3 and CTLA-4 staining density in the four tumor regions and performed counting under 400× magnification.	with high numbers of $FoxP3^{+}$ T-cells in the parenchyma of the invasive front.		
Quan et al.[68] China	159	66 (tongue); 45 (buccal mucosa); 30 (gingiva); 18 (floor of mouth)	139 (M); 20 (F)	52 years (mean)	74 (I/II); 85 (III/IV)	NI	NI	144 (well differentiated);15 (moderate or poor differentiated)	48 months (mean)	T cells: CD4, CD8 / B cells: CD20, CD138 / Treg: FoxP3	For each slide, five representative fields were selected for initial screening under a Leica light microscope at low power (100 ×). Then, we counted the numbers of each type of immune cell at high power (400 ×). The final density of each section was calculated as the average number of five high-power fields (HPFs). The location of TILs was defined as tumor stroma between tumor nests.	Higher expression levels of Foxp3+ and CD138+ TIL were positively associated with poor prognosis	All checklists were completed	Moderate
Li et al.[62] China	168	73 (tongue); 39 (buccal); 37 (gingiva); 19 (others)	120 (M); 48 (F)	79 (≤57 years); 89 (>57 years)	NI	NI	NI	103 (high); 65 (medium/low)	5 years	T cells: CD8 / NK cell: CD57	Briefly, 10 representative fields of the tumour tissue (×40 objective). The total number of each immune cell type was counted in the tumour stroma, excluding those within tumour nests. The average number of 10 fields was calculated as the final density of each section (cells per field).	In multivariate analyses, CD8 expression was independent prognostic factors for both the 5-year OS rate (HR = 2.301; 95% CI, 1.237–4.281) and RFS rate (HR = 2.467, 95% CI, 1.178–4.731). CD57 expression was independent prognostic factor for 5-year OS (HR = 1.579, 95% CI, 1.190–3.154).	Checklist no. 2 was not fulfilled	Low
Ni et al.[36] China	153	Tongue	74 (M); 79 (F)	56,4 years (mean)	71 (I/II); 82 (III/IV)	NI	41	60 (well differentiated); 93 (medium to poor	NI	T cells: CD3, CD4, CD8 / Treg: FoxP3 / DC: CD1a	Each counted the number of cells located in tumor nest and tumor stroma in 5 randomly selected high-power fields (400×). The median was defined as	Patients with higher CD1a+ DCs had better OS. In conclusion, tumor-infiltrating CD1a+ DCs was associated	All checklists were completed	Low

								differentiated)			cutoff value, which was 60, 22, 26, 10, 15, 0.73 and 2.52 for CD3, CD4, CD8, FoxP3, CD1a, CD4/CD8 and CD8/FoxP3, respectively. Low tumor infiltration was defined as score below the cutoff value, while high tumor infiltration was defined as score equal to or above the cutoff value.	with favorable clinical outcomes but not independent prognostic factors for TSCC patients.		
Sales de Sá et al.[23] Brazil	48	Tongue	35 (M); 13 (F)	61,2 years (mean)	13 (I); 10 (II); 8 (III); 17 (IV)	17	10	16 (well differentiated); 25 (moderately differentiated); 7 (poorly differentiated)	NI	T cells (CD3+, CD4+ e CD8+), B cells (CD20+), Dendritic cells (immature (CD1a)) and mature (CD83), Macrophages (CD68)	Quantification of positive dendritic cells (CD83 and CD1a) the individual value was the average of the total of 10 positive cell fields using ImageJ software (version 2.0). The density of CD3, CD4, CD8, CD20, and CD68 expression was scored using semiquantitative score based on the percentage of positive immune cells was scored as (1) negative (<5%), (2) weak (5–30%), (3) moderate (30–80%), and (4) strong (>80%) for each field. The optimal cut-points for cell surface markers were selected to enable sorting into low and high expression groups.	The high expression of CD3+ T cells and B cells are predictive of better overall survival and indicative of an immunologically active, inflammatory tumor immune microenvironment (TIME) in patients with better survival. The number of CD3+ T cells was an independent prognostic marker.	All checklists were completed	Low
Lequerica-Fernández et al.[27] Spain	125	51,41% (tongue); 37,30% (mouth floor); 22,18% (gingiva); 7,6% (buccal mucosa); 6,4% (retromolar area); 2,1% (palate)	82 (M); 43 (F)	58.69 years (mean)	20 -16% (I); 32 - 26% (II); 26 - 20% (III); 47 - 38% (IV)	54	51	80 - 64% (well differentiated); 41 - 33% (moderate differentiated); 4 - 3% (poorly differentiated)	74.10 months (mean)	T cells: CD4, CD8; Treg: FoxP3	CD4, CD8 and FOXP3 immunostainings in both the tumor nests and the surrounding stroma were scored using the average of positively stained cells in each 1 mm2 area from three independent high-power representative microscopic fields (HPFs, 400×; 0.0625 µm2). The CD8/FOXP3 ratio was calculated using the CD8 and FOXP3 results. Since the cut-off value for TILs has not been standardized, the median of the number of CD4+, CD8+ and FOXP3+ cells/mm2 was chosen and used for survival analysis with	Infiltration of stromal/tumoral FOXP3+ TILs was significantly associated with better disease-specific survival. TILs may act as biomarkers and potential therapeutic targets for OSCC.	All checklists were completed	Low

											patients categorized into high (above the median) and low (below the median) subgroups.			
Kikuchi et al.[15] Japan	103	59 (tongue); 26 (gingiva); 9 (oral floor); 8 (buccal); 1 (hard palate)	60 (M); 43 (F)	70 years (median)	35 (I); 32 (II); 10 (III); 26 (IV)	NI	18	NR	40.8 months (median)	Lymphocytes: CD3, CD4, CD8; Macrophage: CD68	Three areas, each of which were 0.55 mm in diameter (equivalent to a 400× magnification in a high-power field; HPF) and represented the three most densely immunocyte-infiltrated areas, were randomly chosen from the field, and cells were counted manually. The total number of immune-marker-positive TAICs was counted, and the average number of TAICs per HPF was calculated for each patient.	Multivariate analysis results adjusted by the pathological stage, resection margin, and extracapsular extension showed that a high number of intratumoral CD68+ were independent negative prognostic markers (hazard ratio: 4.15; P = .01). cD68+ tumor-associated immune cells (TAICs) in the intratumoral area could act as novel biomarker for predicting overall survival outcomes in OSCC patients.	All checklists were completed	Low
Huang et al.[61] China	80	56 (tongue); 10 (buccal); 7 (gingiva); 3 (maxilla); 4 (mouth floor)	47 (M); 33 (F)	54 (<60 years); 26 (≥60 years)	NI	13	14	50 (well differentiated); 30 (moderate and poor differentiated)	66 months	CD8 (T cells), CD20 (B cells), CD57 (NK cells), CD163 (macrophages)	Firstly, we screened the samples under a microscope at ×100 magnification, and five fields showing the highest infiltration of CD8+, CD57+, CD163+, and CD20+ TIICs were obtained, and each field was magnified to ×400 to count the number of TIICs manually. And the mean of area analyzed was roughly 0.1 mm2.	High infiltration of CD57+ NK cells and CD20+ B cells indicate a favorable OS in clinical early-stage OSCC. The nomogram constructed based on TIICs might be used for predicting the prognosis in clinical early-stage OSCC.	All checklists were completed	Low
Ito et al.[22] Japan	33	17 (tongue); 3 (maxillary gingiva); 1 (buccal mucosa); 2 (plantar of the mouth)	13 (M); 20 (F)	66.2 years (mean)	4 (I); 14 (II); 3 (III), 10 (IV A); 2 (IV B)	22	NI	NR	6 months (minimum)	Lymphocytes:CD3, CD8, CD45RO	TILs were evaluated as lymphocytes in the center of the tumor (CT) and at the invasive margin (IM). At 400x magnification, three fields were selected for counting the positive cells. The samples were classified in order of cell density: the top 50% of cases were designated as the TILs High group, and the bottom 50% were designated as the TILs Low group in the CT and IM regions.	No statistically significant associations of CD3 and CD8 with patients' OS and DFS were found. The disease-free survival (DFS) rate of the CD45RO+/TILsHigh group was significantly greater than that of the CD45RO+/TILsLow group in both the CT (low vs. high: mean 64.4 vs. 125.9	Checklist no. 2 was not fulfilled	Moderate

months; p = 0.0045) and IM (low vs. high: mean 10.4 vs. 80.9 months; p = 0.0003) regions (Figure 3). The OS rate of the CD45RO+/TILsHigh group was significantly higher than that of the CD45RO+/TILsLow group on both CT (low vs. high: mean 64.4 vs. 125.9 months; p = 0.0007) and IM (low vs. high: mean 55.4 vs. 158.7 months; p = 0.0031) areas (Figure 4). CD45RO+/TILsHigh in the IM area demonstrated a significant correlation for the DFS in multivariate analysis. In addition, the hazard ratio of the CD45RO+/TILsHigh group in both the CT and IM areas exhibited a significant correlation with the OS in multivariate.

Legends: UK: United Kingdom; USA: United States of America; M: male; F: female; NI: not informed; NR: not realized; RT: radiotherapy.
