# Supplementary material for: Prognostic Value of the Immunohistochemical Detection of Cellular Components of the Tumor Microenvironment in Oral Squamous Cell Carcinoma: A Systematic Review
Source: Curr Issues Mol Biol. 2025 Jul 12;47(7):544. doi: 10.3390/cimb47070544 (PMC12293956; doi:10.3390/cimb47070544)
Supplement: Supplementary file 1 [file cimb-47-00544-s001.zip › Supplementary material S6.pdf]

Supplementary material S6. Summary of descriptive characteristics, analysis methods and results of included studies that evaluated Angiogenesis.

Author, publication year and country	Sample size	Sublocation of oral cavity tumor	Sex	Age	TNM staging	Local recurrence	Death	Histological grading (WHO)	Follow-up	Biomarker used	IMH analysis method	Conclusions/Main results	Compliance to REMARK guidelines	Risk of Bias (MAStARI)
Zhao et al.[48] China	86	13 (mouth floor); 39 (tongue); 22 (cheek); 12 (gingival)	59 (M); 27 (F)	56 years (mean)	18 (I); 33 (II); 9 (III); 26 (IV)	44	29	55 (well-differentiated); 25 (moderately differentiated); 6 (poorly differentiated)	31 months (mean)	D2-40	Under a microscope, three optical fields with the highest lymphatic vessel density [so-called hot spots' (27)] were identified in each sample at ·40 magnification. Lymphatic vessels were counted using ·200 magnification. Lymphatic vessel density (LVD) was defined as the number of lymphatic vessels per optical field, corresponding to an examination area of 0.7386 mm2.	Lymphangiogenesis indeed occurs in oral squamous cell carcinoma; intratumoral lymphatic density might be used as an index to inflect the aggression of the disease, to evaluate the status of lymphatic metastasis, to separate patients at higher risk of an adverse clinical outcome.	Checklists no. 4, 5 and 6 were not fulfilled	High
Chung et al.[66] Korea	62	Tongue	37 (M); 25 (F)	55 years (median)	23 (I); 11 (II); 11 (III); 17 (IV)	NI	NI	43 (well differentiated); 19 (moderate/ poor differentiated)	43 months (median)	D2-40, CD34	Lymphatic vessel density was defined as the number of lymphatic vessels per high-magnification field (x100). At first, the entire section was scanned for the expression pattern of podoplanin, the tumor boundary, and the distribution of lymphatic vessels at low magnification (x40). Three areas of tissue (''hot spot'') with distinctly increased numbers of lymphatic vessels were then selected from inside the tumor and areas within a distance of 500 lm from the tumor periphery, respectively. All immunostained vessels in each hot spot were counted at high magnification (x100).	Intratumoral lymphatic densities showed a strong correlation with regional metastasis in patients with squamous cell carcinoma of the tongue.	Checklist no. 5 was not fulfilled	Moderate
Wirsing et al.[39] Norway	75	35 (mobile tongue); 40 (all others)	43 (M); 32 (F)	0–59 (n=28) ≥60 (n=47)	NI	NI	NI	28 (well differentiated); 42 (moderate differentiated)	10 years (minimum)	PNAd	Micrographs of the five areas with highest HEV density were taken with a Leica DFC 420 camera on a Leica DM2000 microscope (Leica, Wetzlar, Germany) at high power magnification (400×), and the number of	The presence of tumour-associated high-endothelial venules to be an easy to use, robust, and independent positive prognostic factor for patients with oral cancer. Absence of these vessels in	All checklists were completed	Low

											HEVs in the photographs was counted manually.	advanced-stage tumours might identify patients with more aggressive disease.		
Ding et al.[40] China	50	Tongue	25 (M); 25 (F)	53.5 years (mean)	25 (I/II); 25 (III/IV)	21	19	28 (I); 20 (II); 2 (III)	60.34 months (mean)	LYVE-1	We had chosen six areas, which had the greatest number of LYVE-1-positive vessels (called 'hot spots'), and then selected two of the hot spots (9200, using a 0.25 mm²) randomly to calculate the median number. When the number of LYVE-1-positive vessels was higher than the median number, the section was identified as LYVE-1 high expression. When the number of LYVE-1-positive vessels was lower than the median number, the section was identified as LYVE-1 low expression.	There was no correlation between the expression of LYVE-1 and overall survival of patients with OTSCC.	All checklists were completed	Low
Seppälä et al.[65] Finland	61	Tongue	33 (M); 28 (F)	61 years (mean)	NI	29	27	24 (I); 25 (II); 12 (III)	5 years	D2-40, vWF	In the tongue cancer specimens, the vessel density was calculated in intratumoral area and whole tumor (including intra- and peritumoral) from five microscopic fields (0.785mm2/field). The relative density of lymphatic vessels (RDLV)was counted by dividing themean number of lymphatic vessels per microscopic field (podoplanin) by the mean number of all vessels (vWf) per microscopic field. vWF is weakly expressed on lymphatic endothelium. Therefore, the positive lymphatic vessel staining of vWfwas assured by the lack of erythrocyte or smooth muscle within the lymphatic vessel walls. Analysis was performed at 200x magnification. The mean diameters of lymphatic vessels were determined in five fields with 400 (40x objective	The lymphatic vessel density and diameter of lymphatic vessels were not associated with tongue cancer survival. The relative density of lymphatic vessels might have clinically relevant prognostic impact. Further studies with increased number of patients are needed.	Checklists no. 2 and 5 were not fulfilled	Low

											lens and 10x ocular lens magnification in intratumoral area and whole tumor. The mean diameter of the samples was graded as S<50lm, M=51–130lm,L<130lm.			
Mafra et al.[56] Brazil	56	Tongue	39 (M); 17 (F)	62 years (mean)	20 (I/II); 36 (III/IV)	14	28	NR	NI	D2-40	D2-40-positive endothelial cells or clusters of these cells with or without a clearly visible lumen were counted as individual microvessels (scale = 100 μm). Lymphatic vessel density was established as the mean number of lymphatic vessels per intratumoral (I-LVD) or peritumoral (P-LVD) microscopic field.	Intratumoral LVD was higher in advanced clinical stages (III/IV) when compared to early-stage (p=0.017) and in metastatic cases compared to non-metastatic tumors (p=0.013). Peritumoral LVD and intratumoral or peritumoral MCD did not differ significantly according to the clinicopathological parameters of OTSCCs (p>0.05). No significant correlations between LVD and MCD were observed at the intratumoral (r=-0.014; p=0.918) or peritumoral level (r=0.156; p=0.251).	Checklists no. 4 and 5 were not fulfilled	Moderate
Wirsing et al.[51] Norway	75	36 (tongue); 21 (floor of the mouth); 9 (alveolar ridge); 7 (buccal mucosa); 2 (others)	43 (M); 32 (F)	28 (0-59 years); 47 (≥ 60 years)	NI	NI	NI	28 (well differentiated); 42 (moderate differentiated); 5 (poor differentiated)	60 months (maximum)	PNAd, D2-40	Two trained, independent observers who were blinded to the clinical outcome evaluated the immunohistochemical staining quantitatively and semiquantitatively.	Vessels are a potential immunomodulatory target in this type of cancer.	All checklists were completed	Low

Legends: UK: United Kingdom; USA: United States of America; M: male; F: female; NI: not informed; NR: not realized; RT: radiotherapy.
